# Efficacy of Synchronous Remote-Based Interventions for Suicide Prevention among Adolescent and Adult Patients: A Systematic Review and Meta-Analysis

**DOI:** 10.1192/j.eurpsy.2022.754

**Published:** 2022-09-01

**Authors:** L. Comendador Vázquez, A. Cebrià Meca, A. Sanz Ruíz, M.P. Jiménez-Villamizar, J.P. Sanabria-Mazo, C. Mateo Canedo, D. Palao Vidal

**Affiliations:** 1 Parc Tauli-University Hospital, Mental Health, Sabadell (Barcelona), Spain; 2 Centro de Investigación Biomédica en Red de Salud Mental (CIBERSAM), Salud Mental, Madrid, Spain; 3 Autonomous University of Barcelona (UAB), Department Of Psychiatry And Legal Medicine, Cerdanyola del Vallès (Barcelona), Spain; 4 Autonomous University of Barcelona (UAB), Department Of Clinical And Health Psychology, Cerdanyola del Vallès (Barcelona), Spain; 5 Autonomous University of Barcelona (UAB), Department Of Basics, Developmental And Educational Psychology, Cerdanyola del Vallès (Barcelona), Spain; 6 School of Medicine, Universitat Autònoma de Barcelona, Medicine, Cerdanyola del Vallés, Spain; 7 Consorci Corporació Sanitària Parc Taulí, Psychiatry, Sabadell, Spain; 8 Centro de Investigaci ´on Biom ´edica en Red de Salud Mental (CIBERSAM), Salud Mental, Madrid, Spain

**Keywords:** Suicide, Emergency Department, e-mental health, secondary prevention

## Abstract

**Introduction:**

Suicide is a universal, complex, and multifaceted public health problem that is among the leading causes of preventable death worldwide. The impact of suicide affects families, communities, and societies; hence its prevention is an emerging priority for public health systems.

**Objectives:**

The current systematic review aims to investigate the efficacy of distance suicide prevention strategies implemented through synchronous technology-based interventions (i.e., any digital tool that allows interactive and immediate real-time communication conducted remotely).

**Methods:**

The bibliographic search has been carried out in the electronic databases MEDLINE/PubMed, PsycInfo, Scopus, and Web of Science, with no restrictions on the publication period and limited to publications in English or Spanish. Two reviewers independently will conduct screenings, data extraction, risk of bias (RoB), and methodological quality assessment.

**Results:**

The preliminary data searches seem to support the effectiveness of providing active contact to persons who have made a suicide attempt and indicate that receiving early specialized assistance decreases the relative risk of recurrence. The reduction would be attributable to improved detection of patients at increased risk and effective referral to emergency services.

**Conclusions:**

Telematics suicide prevention has been an emergent field for years, facilitated by the notably increased in acceptance and availability. Considering that distance programmes can reach affected individuals regardless of their location, it stands to reason that these interventions will be part of future suicide prevention efforts. The results will be discussed regarding (a) the effect size of the intervention outcomes and (b) the main moderators of the effectiveness found.

**Disclosure:**

No significant relationships.

